# Dosimetry of a novel focused monoenergetic beam for radiotherapy

**DOI:** 10.1088/1361-6560/ac5c8f

**Published:** 2022-03-29

**Authors:** Jamshid Moradi-Kurdestany, Dirk A Bartkoski, Ramesh Tailor, Dragan Mirkovic, Ze’ev Harel, Aharon Bar-David, Michael Kleckner, Shirly Borukhin, Mohammad Salehpour

**Affiliations:** 1 Department of Radiation Physics, The University of Texas MD Anderson Cancer Center, Houston, TX, United States of America; 2 Convergent Radiotherapy and Radiosurgery, Tirat Carmel, Israel

**Keywords:** gafchromic film dosimetry, energy response, orthovoltage dosimetry

## Abstract

*Objective*. A novel treatment modality is currently being developed that produces converging monoenergetic x-rays. Conventional application of dosimetric calibration as presented in protocol TG61 is not applicable. Furthermore, the dosimetry of the focal point of the converging beam is on the order of a few millimeters, requiring a high-resolution dosimeter. Here we present a procedure to calibrate radiochromic film for narrow-beam monoenergetic 60 keV photons as well as absolute dosimetry of monoenergetic focused x-rays. A study of the focal spot dose rate after passing through a bone-equivalent material was also done to quantify the effects of heterogeneous materials. *Approach.* This was accomplished by configuring a polyenergetic beam of equivalent energy using a clinical orthovoltage machine. Calibrated films were then used to perform absolute dosimetry of the converging beam by measuring the beam profile at various depths in water*. Main Results.* A method for calibrating radiochromic film has been developed and detailed that allows absolute dosimetry of a monoenergetic photon beam. Absolute dosimetry of a focused, mono-energetic beam resulted in a focal spot dose rate of ∼30 cGy min^−1^ at a depth of 5 cm in water. *Significance.* This work serves to establish a dosimetry protocol for mono-energetic beam absolute dosimetry as well as the use of such a method for measurement of a novel teletherapy modality.

## 1. Introduction

**Figure 1.**
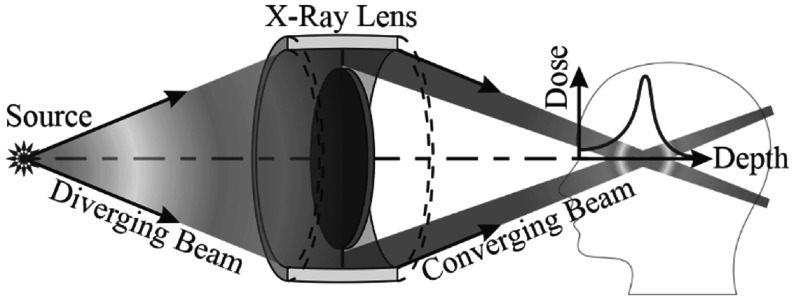
Cross-sectional conceptual representation of a Bragg reflecting lens. A polyenergetic diverging beam, such as produced by an x-ray tube, is reflected from a lens structure using Bragg refraction with the central non-reflecting portion of the beam being blocked. The result is a converging monoenergetic beam with a highly localized dose distribution at the focal point. Reproduced from Bartkoski *et al* (2021), with permission from Springer Nature. CC BY 4.0.

A novel treatment modality currently in development at the University of Texas MD Anderson Cancer Center in collaboration with Convergent Radiotherapy and Radiosurgery (CRnR) has the potential of providing highly localized dose distributions with enhanced healthy tissue sparing. By using an annular configuration of aluminum single-crystal tiles, a diverging beam of x-rays may be converted to a converging beam with a focal spot size of a few millimeters, resulting in high-intensity localized dose distributions as illustrated in figure 1.

Bragg refraction, the process by which x-rays are reflected from metallic crystals, produces a nearly monoenergetic output spectrum from a polyenergetic input beam. The resulting conically shaped, converging, quasi-monoenergetic 60 keV photon beam, composed primarily of the K_1_ *α* and K*α*_2_ tungsten characteristic x-rays surrounded by an ∼8 keV band x-ray energies, is unlike that of conventional teletherapy beams. A detailed description of the CRnR converging beam system including a discussion of physics principles and initial prototype measurements results may be found in Bartkoski *et al* (2021).

Robotic manipulation of the delivery system will allow for the focal spot to be ‘painted’ into 3D conformal doses. The unique combination of properties afforded by the CRnR beam make it ideal for tumors adjacent to highly radiosensitive structures such as the spinal column. Head and neck tumors also have the potential to benefit from the converging beam geometry as well as being a tool for stereotactic radiosurgery. Additionally, non-cancer related treatments are being considered such as age-related macular degeneration and denervation which require highly localized radiation delivery.

Physics aspects such as dose calibration, dosimetric measurements, and treatment planning of such a treatment modality as produced by the CRnR system present unique challenges. Dosimetry requirements for this beam include good spatial resolution, low energy dependence, and negligible dose-rate dependence. Radiographic EBT3 film appears to be a good candidate for absolute dosimetry. However, its dose-calibration protocol requires an Accredited Dosimetry Calibration Laboratory (ADCL)-calibrated ion chamber, preferably with low energy dependence. The use of radiochromic films for medical and industrial applications has been established and exhaustively discussed (Butson *et al* 2003, Butson *et al* 2004, Schwob and Orion 2007, Williams and Metcalfe 2011, Niroomand-Rad *et al* 2020). In this work, we describe a method for calibrating radiographic EBT3 film for a narrow-beam geometry, monoenergetic x-ray beam equivalent to the beam produced by the CRnR system. The calibrated films were then used to perform a variety of absolute dose measurements. These absolute dose measurements are used to further analyze the dose deposition characteristics of the CRnR beam. Relative dosimetery analysis of this unique beam has been reported in Bartkoski *et al* (2021).

## 2. Methods

### 2.1. Equivalent polyenergetic beam

Current ADCL measurements do not include dose calibration for monoenergetic beams. Therefore, an equivalent polyenergetic beam was needed. A polyenergetic beam may be substituted for a monoenergetic beam by using the half-value layer (HVL). The HVL is the thickness at which half of the original photon intensity of a monoenergetic beam is attenuated by a material with attenuation coefficient *μ* and is given by

\begin{eqnarray*}{\mathrm{HVL}}=\displaystyle \frac{{\mathrm{In}}\,2}{\mu }.\end{eqnarray*}

A polyenergetic beam is said to be approximately equivalent to a monoenergetic beam with the same first HVL. Furthermore, for a monoenergetic beam, the first and second HVLs would be equal but beam hardening in polyenergetic beams result in longer second HVLs. The homogeneity factor (HF) is the ratio of the first to second HVL and indicates how closely a polyenergetic beam approximates a monoenergtic beam with ${\mathrm{HF}}=1.$ The mass attenuation coefficient of *μ/ρ* = 0.2778 cm^2^ g^−1^ (Hubbell and Seltzer 1995) for aluminum with density *ρ* = 2.699 g cm^−3^ for a 60 keV mononenergetic beam results in an HVL of 9.2 mm.

The polyenergetic beam was configured on an Xstrahl 300 orthovoltage unit in narrow-beam geometry using a 180 kVp beam energy and corresponding filter No. 6 as specified by the manufacturer. Filter No. 6, which includes 1.5 mm Al and 0.15 mmCu, was modified with an additional 1 mm Al such that a voltage of 180 kVp at 15 mA produced the necessary attenuation to produce a first HVL = 9.0 mm and a measured HF of 0.76.

The process for calibrating film for a monoenergetic beam is as follows:

1. An ion chamber is sent to ADCL for calibration at polyenergetic beam energies (*E*_1_ and *E*_2_) close to the desired beam energy *E*_0_.
2. The ion chamber calibration is interpolated at *E*_0_ from *E*_1_ and *E*_2_.
3. Using the aforementioned configured polyenergetic beam, a specified amount of radiation is delivered to the ion chamber, and the dose is recorded.
4. Using the same configuration, the same amount of radiation is delivered to the film, and the film’s optical density (OD) is recorded.
5. The process is repeated for varying amounts of radiation delivered, and a calibration curve of dose versus OD is generated.

The film calibration was justified for a monoenergetic beam by repeating the film calibration process using the polyenergetic beam at various neighboring energies around the monoenergetic energy *E*_0_. A negligible energy dependence of the dose versus OD calibration curves justified the usage for a monoenergetic source.

### 2.2. Dosimeter selection

With an inherent spatial resolution <25 *μ*m, dose rate dependence <5%, and an energy dependence <5%, the near-tissue-equivalent Gafchromic EBT3 film (lot No. 07281401, Ashland Inc., Wayne, NJ, USA) was chosen for absolute dosimetry of the converging beam focal spot. An Epson 10000XL flatbed scanner was employed in transmission mode to read the exposed film pieces. The scanner used 48-bit color with a spatial resolution of 72 dpi and was chosen because the scanner is the limiting factor in contributing to film resolution.

A Farmer-type ion chamber (NEL 2515/3, serial No. 2402) was used as the ADCL-calibrated dosimeter. Ion chamber calibration coefficient *N*_*k*_ were obtained in terms of air kerma from the ADCL for 100, 125, and 250 kVp from which a chamber calibration coefficient for 180 kVp was interpolated (see table 1).

**Table 1.** Beam energy, first half value layer, and ion chamber calibration coefficients *N*_*k*_ for three ADCL beams obtained for ion chamber NEL2515/3 as well as the interpolated *N*_*k*_ at 180 kVp.

Beam (kVp)	HVL (mmAl)	ADCL *N*_*k*_ (cGy/nC)	Interp *N*_*k*_ (cGy/nC)
100	4.14	11.84	—
125	6.01	11.87	—
180	9.0	—	12.01
250	17.8	12.42	—

### 2.3. Film calibration

EBT3 cross-calibrations were performed for three orthovoltage energies, 125 kVp (20 mA, filter No. 3, HVL 3.5 mm Al), 180 kVp (15 mA, modified filter No. 6, HVL 9.0 mm Al), and 250 kVp (10 mA, filter No. 7, HVL 1.3 mm Cu). Calibration protocol TG61 was employed (Ma *et al* 2001) to calibrate the beams output for dose to water in air. The following equation from TG-61 gives the absorbed dose to water in air at the phantom surface for low and medium energy x-rays

\begin{eqnarray*}{D}_{w,surface\,}=M{N}_{K\,}{B}_{W}{P}_{stem,air}{\left[{\left(\displaystyle \frac{{\bar{{\mu }}}_{{\mathrm{en}}}}{\rho }\right)}_{air}^{w}\right]}_{air},\end{eqnarray*}

where *M* is the free-in-air ion chamber reading corrected for temperature, pressure, ion recombination, and electrometer accuracy. For a given beam quality, ${N}_{K}$ is the ADCL-provided air-kerma calibration coefficient; the chamber stem correction factor ${P}_{{stem},{air}}$ was set to unity as the measurement field size was set to the calibration field size, and ${\left[{\left({\bar{\mu }}_{{en}}/\rho \right)}_{{air}}^{w}\right]}_{{air}}$ is the water-to-air ratio of the mean mass energy-absorption coefficients averaged over the incident photon spectrum. The backscatter factor ${B}_{w},$ which accounts for scatter from the phantom, was neglected due to the EBT3 film pieces being suspended in air on a 0.01 mm thick layer of polyethylene stretched over a 15 × 15 cm polystyrene foam frame. This allows the calculation of dose to a water medium in air, represented by the suspended water-equivalent EBT3 film.

A sheet of EBT3 was cut into 50 pieces, each 2 cm × 2 cm. Reading an exposed film is known to exhibit angular-dependence due to polarization effects (Butson *et al* 2003); therefore, it is important to maintain the grain orientation while scanning film pieces. To achieve this, before cutting the parent film into pieces, ink lines were marked to show 2 cm × 2 cm pieces, and ID numbers were marked consistently on bottom right corners. Film pieces were laid on the scanner bed maintaining the ID number orientation in a consistent manner.

Each film piece was irradiated in air to a known dose in reference geometry (field size 10 cm × 10 cm, source-to-film distance 50 cm). Seven dose levels were used, ranging from 0 to 1200 cGy. For each dose level, two film pieces (one at a time) were exposed to serve as repeat data. The same procedure was employed for each of the three beam energies.

### 2.4. Reading exposed films

Exposed EBT3 is known to exhibit an OD that continues to develop over time (Khachonkham *et al* 2018). Therefore, all exposed pieces were read after 1 d (±2 h).

To obtain OD, each film piece was scanned before and after exposure. Two requirements are needed in order to achieve the highest possible OD precision. First, film grain orientation should be the same for all pieces, which is achieved by maintaining film ID number orientation. Second, a film piece should go to the same physical location on the scanner glass for pre- and post-exposure scans. This is achieved by employing a cardboard template with a cut-out window for placement of the film pieces. Each film ID number sits in the template window same way for pre- and post-exposure scans.

Three repeat scans were acquired for pre- and post-exposed films. All images were saved in TIFF format. TIFF images are read into the ImageJ software (developed at the U.S. National Institutes of Health and available at http://rsb.info.nih.gov/nih-image/) in RGB format, where the red channel is used for analysis. A region of interest (ROI) 0.5 cm × 0.5 cm at the center of each scan was employed to obtain the ROI mean pixel value representing the transmitted light intensity. This value along with the ROI standard deviation was recorded where the standard deviation is used to propagate error.

### 2.5. Transmitted intensity to OD conversion

Using the notations *I*_*pre*_ and *I*_*post*_, respectively, for the films’ pre- and post-exposure transmitted light intensity, net optical density ${NetOD}$ was calculated using the following equation (Devic *et al* 2004)

\begin{eqnarray*}NetOD={lo}{{g}}_{10}\left(\displaystyle \frac{{I}_{pre}-{I}_{opq}}{{I}_{post}-{I}_{opq}}\right)\end{eqnarray*}

*I*_*opq*_, a scanner calibration factor that considers the light scattered into the scanner that can reach the irradiated film, is acquired by doing a blank scan with the scanner bed covered in black cloth. Film calibration curves report dose delivered in cGy versus net optical density.

### 2.6. Absolute dosimetry techniques

Films were irradiated in two different orientations. Transverse, or horizontal, films, where irradiated such that the plane of the film was perpendicular to the beam axis while longitudinal, or vertical, films were parallel to the beam axis. Vertical films were aligned such that the plane of the film passed through the beam’s focal spot. Horizontal films were placed at different positions along the beam path depending on the desired beam cross-section. Measurements were performed in air as well as in a 30 × 15 × 20 cm water-filled phantom.

The tissue-maximum ratio is the ratio of dose or dose rate to the maximum dose and gives a quantitative measure of how dose deposition changes with depth in the target volume. Since the focal spot was fixed in space, the depth in water was varied by moving the phantom longitudinally relative to the focal spot. In this manner, focal spot doses were recorded at depths from 0 to 12 cm.

To fully quantify the CRnR beam for treatment planning, it was important understand how the beam interacts with different materials. Polybutylene terephthalate (PBT), a.k.a. Teflon, is often used as bone-equivalent material. A PBT sample was imaged with a Varian cone beam CT scanner at 100 kVp, 80 mAs, to compare the Hounsfield units to that of bone.

Measurements were performed to analyze the effect of beam passage through heterogeneous materials. A stack of materials, starting with the top, consisted of 0.5 cm thick PBT, 5 cm solid water, EBT3 film, and 10 cm solid water were placed in the beam such that the film was at the focal spot. This simulated 5 cm depth in water after passing through 5 mm of bone. The 5 mm of PBT was then replaced with 5 mm of solid water and the measurement repeated.

## 3. Results and discussion

**Figure 2.**
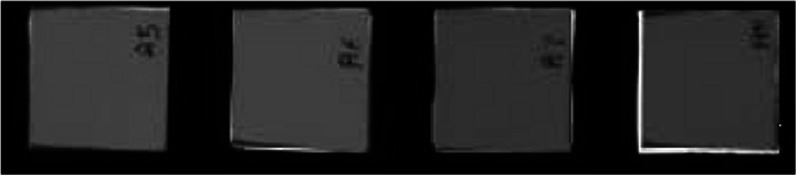
Sample exposed films for dose-calibration. EPSON 10000XL scanner settings where: transmission mode, positive film, 75 dpi, and 48-bit color. The scanned images were analyzed with ImageJ software using red channel. The left two films were both exposed to 150.4 cGy with the right two films being exposed to 300 cGy.

**Figure 3.**
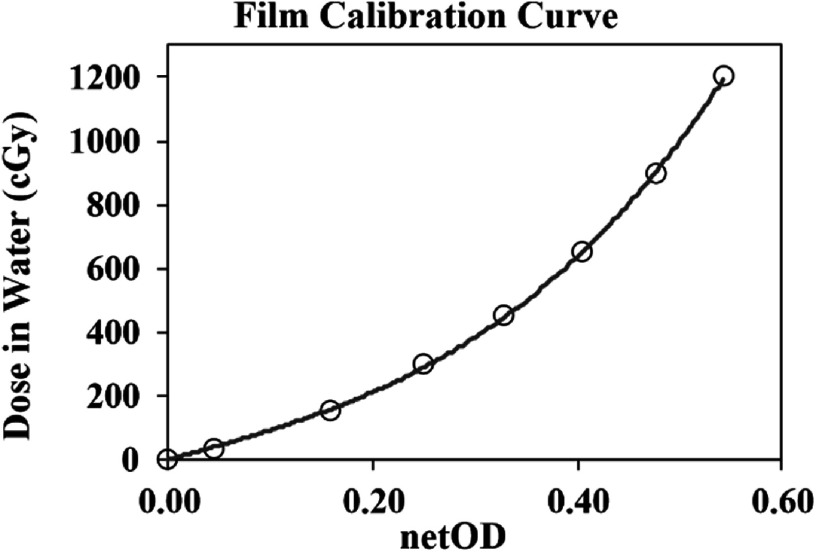
EBT3 calibration curve for an equivalent polyenergetic beam configured using 180 kVp, 15 mA, modified filter No. 6, and HVL 9.0 mm Al. The line represents a third order polynomial fitted to the optical density data.

An example of film pieces exposed to known doses is shown in figures 2, and 3 depicts an EBT3 calibration curve, measured in red channel, for the equivalent polyenergetic beam where the ion chamber was configured using a water-to-air conversion factor of 1.0535 interpolated for HVL 9.0 mmAl from Table IV in Ma *et al* (2001), so dose is presented as dose to a water medium. A third order polynomial, forced to pass through origin, was fitted to the data with the following equation:

\begin{eqnarray*}Dose\left(cGy\right)={a}_{1}\left(netOD\right)+{a}_{2}{\left(netOD\right)}^{2}+{a}_{3}{\left(netOD\right)}^{3}.\end{eqnarray*}

Coefficients ${{a}}_{1},{a}_{2},$ and${a}_{3}$ are 897.6, −123.5, and 4639, respectively. The presented dose-response curve, measured for EBT3 lot No. 07281401, may not apply to different lots. Usage of films from a different lot requires recalibration.

### 3.1. Energy dependence

**Figure 4.**
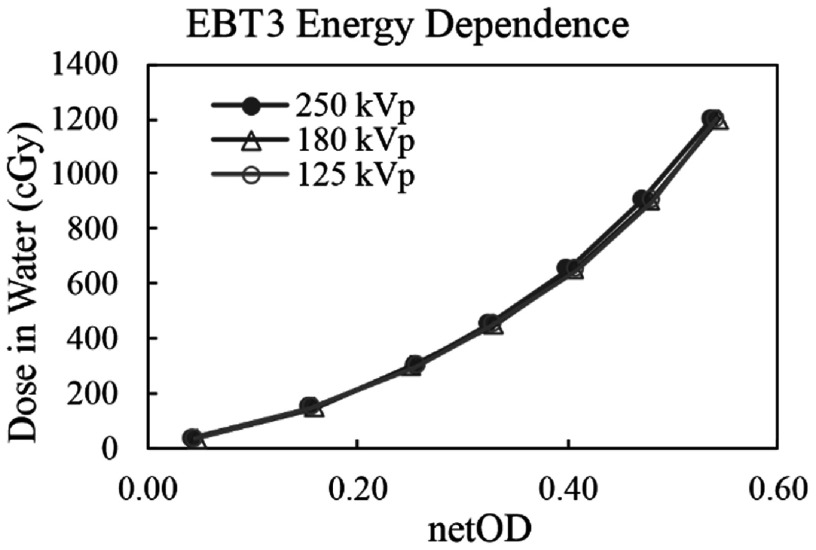
The results show that EBT3 film response is practically independent in the presented energy range, i.e. for a given spatial resolution, negligible difference between beam energies can be seen.

To show energy dependence, dose calibrations at three energies (125 kVp, 180 kVp, and 250 kVp) are presented in figure 4, and the fit coefficient values as well as beam quality parameters are reported in table 2. The curves for the two lower energies are almost indistinguishable and differ by <1%. The 250 kVp curve shows a slight over-response of ∼3% over the 400–1200 cGy region. This discrepancy should be viewed within the perspective of measurement uncertainty, which will be discussed shortly. The minimal energy dependence of EBT3 resulting from measured data for the orthovoltage energy range and dose range of interest has previously been reported for polyenergetic beams (Guerda Massillon *et al* 2016) and monoenergetic beams (Brown *et al* 2012).

**Table 2.** First half value layers and effective energies are shown for each of the beam energies used. Coefficients *a*_1_, *a*_2_, and *a*_3_ of the third order polynomial fit to the data for netOD to dose conversion for the beam energies.

Beam Energy	HVL	Eff. E (keV)	*a*_1_	*a*_2_	*a*_3_
125 kVp	3.5 mmAl	35.5	933.1	−446.7	5147
180 kVp	9.0 mmAl	60.0	897.6	−123.5	4639
250 kVp	1.3 mmCu	89.1	834.3	260.2	4363

### 3.2. Dose measurement uncertainty

Various factors contribute to uncertainty in dose determination from film. A film calibration, as represented in figure 3, is the result of two measured quantities; the calibrated dose delivered to the film and the OD read from the film, each with their own uncertainties. Following is an analysis of the overall uncertainties and contributing factors to each.

### 3.3. OD uncertainty

Several steps were taken to minimize systematic uncertainties in the film results. There was no batch-to-batch variation as a single batch was employed. Light polarization effects were avoided by ensuring consistent film-grain orientation during scanning as previously described. Variation due to film’s self-development was minimized by maintaining the same time difference between radiation exposure and film scanning. Variation due to scanner-bed uniformity was avoided by using a template, which ensured that film pieces were positioned in the same location on the scanner bed.

Uncertainty in OD results from (a) film non-uniformity, (b) film-to-film variation, (c) non-uniformity of scanner-bed response over the region in which film pieces were placed, and (d) drift in scanner response for post- versus pre-exposure scans.

**Figure 5.**
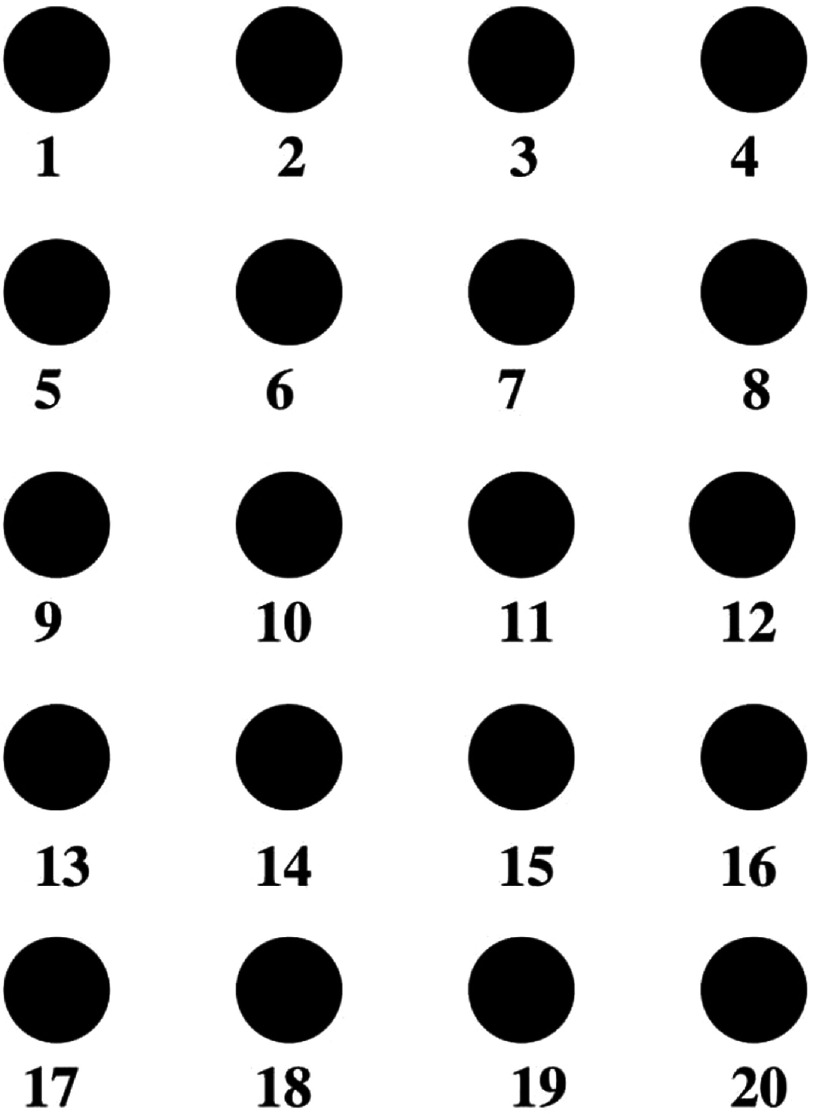
Site pattern used for measurement of EBT3 homogeneity.

**Figure 6.**
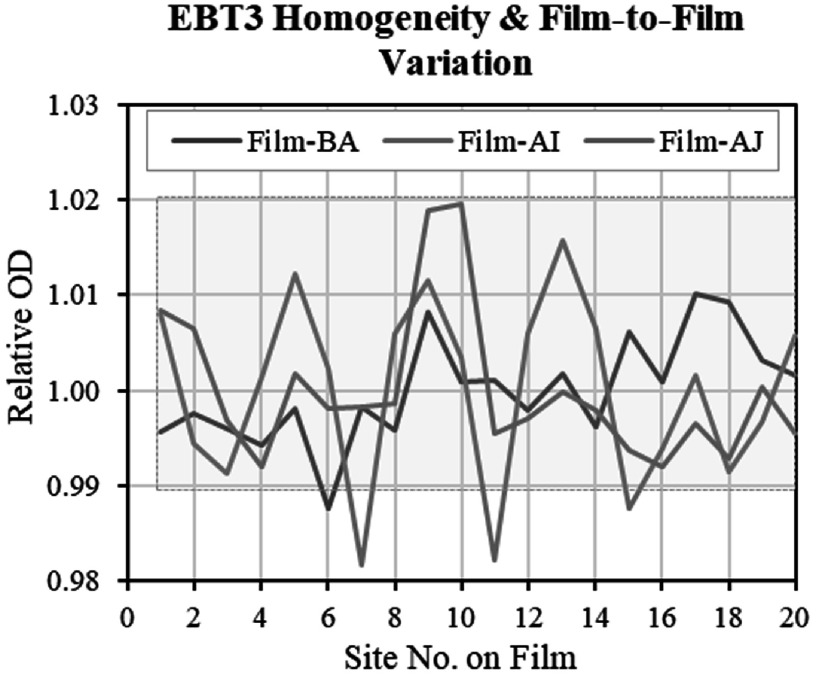
Combed film homogeneity and film-to-film variations for three EBT3 (Lot 10231801) films. Expanded uncertainty (*k* = 2) is shown as a highlighted box around data.

In order to evaluate the film non-uniformity, film-to-film variation, and non-uniformity of scanner-bed response uncertainties, three film sheets were chosen (identified as BA, AI, and AJ) from the same batch used for the calibration procedure. Each film was cut into 20 pieces (see figure 5) and exposed to same dose of 600 cGy with a 180 kVp (15 mA, modified filter No. 6, HVL 9.0 mmAl) beam from an Xstrahl 300 orthovoltage unit. Exposed films were scanned on the Epson 10000XL after the same 24 h waiting period used for film calibration and analyzed with ImageJ using the red channel. Pixel values were converted to OD and then to dose by employing the afore-mentioned film calibration. Results showing relative OD variations are presented in figure 6 which shows and expanded uncertainty (coverage factor *k* = 2) of 1.6%. Scanner-bed non-uniformity was determined to have an uncertainty of 0.8% (*k* = 2). The fourth uncertainty component, the drift in scanner response, was tracked over ∼1 year (data not presented) and was found to be negligible (<0.1%). Added in quadrature, the overall uncertainty in OD with *k* = 2 is 1.8%.

### 3.4. Dose uncertainty

Uncertainty in delivered dose results from ion chamber calibration related uncertainties (a) from the National Institute of Standards and Technology (NIST), (b) ADCL contribution, as well as the (c) electrometer (d) setup uncertainty, and (e) beam output variation. The uncertainty budget for the air kerma calibration coefficient is reported to be 1.50% for the combined contributions of NIST and ADCL with a coverage factor of 2. Electrometer uncertainty in the range of 0.1–700 nC is 0.13%. Setup errors amount to 0.5%. Historical reports of monthly dose-output checks indicate a dose reproducibility of ±0.4% (*k* = 2) (Lim *et al* 2021). Therefore, an overall uncertainty in doses delivered to film pieces is estimated to be 1.64% (*k* = 2). Uncertainty in film-measured dose is expected to be higher at lower doses, as signal OD gets closer to noise, or background OD.

### 3.5. Absolute dosimetry

#### 3.5.1. Tissue-maximum ratio

**Figure 7.**
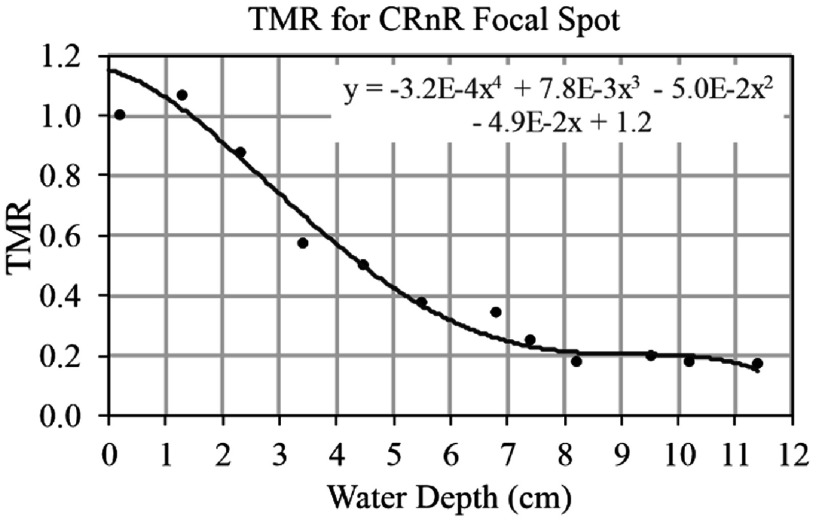
Tissue-maximum ratio (TMR) at the focal spot for various depths for a beam produced with the CRnR system using 125 kVp, 28.6 mA source parameters. Polynomial fit and equation are also shown.

Presented in figure 7 is the tissue-maximum ratio that shows a fairly linear decrease in dose rate in water at depths up to 8 cm. It is expected that the maximal clinical depth for the CRnR system is 7–8 cm in water.

#### 3.5.2. Depth analysis

**Figure 8.**
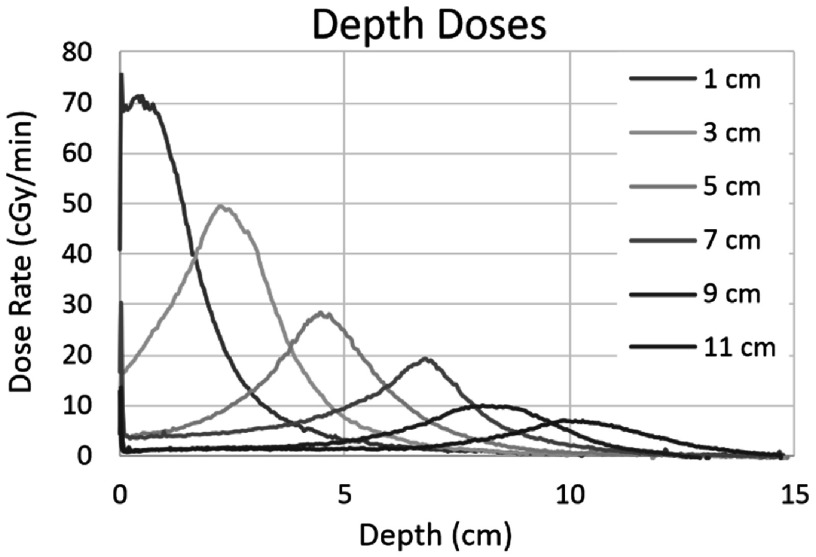
Depth dose curves for focused monoenergetic 60 kV x-rays in water taken longitudinally down the central axis passing through the focal spot where the different curves represent the spot at different depths.

Vertical films were used produce longitudinal profiles of the beam at various depths in water. These measurements were also used to characterize other important aspects of the beam. Central axis depth dose curves are reported in figure 8 and display the evolution of the longitudinal profile of the focal spot at increasing depths in water. Measurement uncertainty results in peaks, i.e. the 11 cm peak, which do not decrease strictly monotonically.

#### 3.5.3. Heterogeneous materials

**Figure 9.**
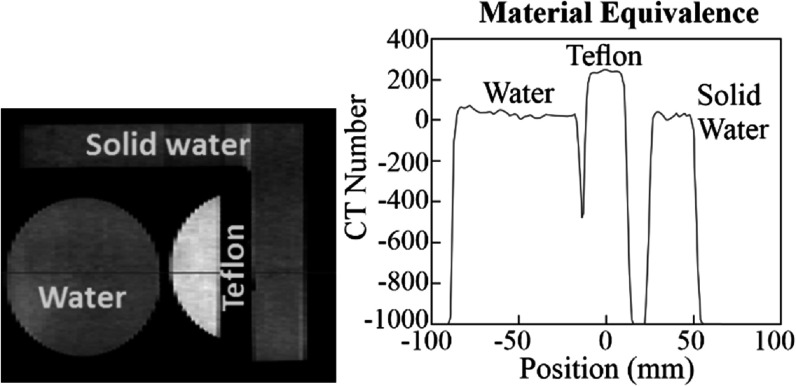
(Left) CT image of a cup of water, polybutylene terephthalate (Teflon), and solid water. CT numbers along the indicated line are plotted. (Right) CT number across the three materials.

Figure 9 shows the CT number comparison between H_2_O, solid water, and PBT. The water and solid water show CT number of ∼0 while the Teflon is ∼225. Bone CT numbers are anywhere from 200 to 1000 (Kalra 2018) meaning that PBT is a sufficient approximation for bone in the CRnR energy range.

The ratio of the focal spot dose rate with the bone-equivalent to that without was 0.87, meaning that passing through 5 mm of PBT upstream of the focal spot attenuated the beam by 13%. A second measurement was performed similar to the first except that the material was stacked in the following order: 5 cm solid water, EBT3 film, 0.5 cm PBT, and 10 cm solid water. The measurement was repeated by replacing the bone-equivalent with 0.5 cm solid water. This measured the enhanced dose on the interface between PBT and tissue as a result of backscatter. The ratio of dose rate with and without the bone-equivalent was 1.2, resulting in an enhanced dose of 20%.

## 4. Conclusions

A method was devised for calibrating EBT3 film for monoenergetic 60 kV focused x-rays. Absolute dosimetry for the CRnR converging beam has been used to evaluate various beam characteristics in various materials. Future work will incorporate these results into analysis of optimal clinical sites for the CRnR system.
